# Commencement of Lectures in University of Buffalo

**Published:** 1862-11

**Authors:** 


*Commencement of Lectures in University of Buffalo.*—Last evening, (Nov. 5th,) the Lecture introductory to the Course, was given by Prof. James P. White, to a large class of students. The occasion was also graced by the attendance of many friends of the College. Of the Lecture we hope to be able to speak hereafter.

